# Glucometer ownership and plasma glucose monitoring practices among persons with type 2 diabetes mellitus on oral antihyperglycaemic therapy in Gulu, Uganda

**DOI:** 10.1097/MD.0000000000042344

**Published:** 2025-05-02

**Authors:** Brenda Nakitto, Winnie Kibone, Ingrid Ampeire, Lauryn Nsenga, Felix Bongomin

**Affiliations:** a Faculty of Medicine, Department of Immunology and Molecular Biology, Gulu University, Gulu City, Uganda; b Department of Immunology and Molecular Biology, School of Biomedical Sciences, College of Health Sciences, Makerere University, Kampala, Uganda.

**Keywords:** glucometer ownership, glucose monitoring, type 2 diabetes mellitus, Uganda

## Abstract

Effective plasma glucose monitoring is crucial for persons with type 2 diabetes mellitus (T2DM) to mitigate long-term complications linked to inadequate glycemic control. This study investigated glucometer ownership and monitoring practices among persons with T2DM in a resource-limited setting in northern Uganda. A feasibility cross-sectional study was conducted, sequentially enrolling T2DM patients on oral antihyperglycemic agents who received routine care at the diabetes clinic of Gulu Regional Referral Hospital from June to August 2023. Data on sociodemographic characteristics, glucometer ownership, glucose monitoring, T2DM treatment, and complications were collected using an interviewer-administered, structured questionnaire. Among 190 eligible participants, with a mean age of 55 ± 12.72 years, 121 (63.7%) were female, 133 (70.0%, n = 133) had primary education, and 160 (84.2%) were unemployed. The median T2DM duration was 4 (IQR: 2–8) years. Treatment included a combination of glibenclamide and metformin for 132 (70.6%) participants. Only 17 (9.0%) owned a glucometer, and 173 (92%) solely measured blood sugars at clinic reviews. Additionally, 173 (91.1%) self-reported at least one DM complication, notably hypertension (n = 121, 63.7%). Tertiary education was independently associated with good glucose monitoring practices (adjusted prevalence ratio: 4.48; 95% confidence interval: 1.21–16.67; *P* = .025). In the diabetes clinic at Gulu Regional Referral Hospital, merely 1 in 10 T2DM patients owned a glucometer. The predominant reliance on clinic-based measurements and the high prevalence of self-reported complications, especially hypertension, underscore the need for targeted interventions to encourage regular home monitoring and enhance overall glycemic control. The observed association between tertiary education and improved glucose monitoring practices suggests that educational initiatives could be pivotal in fostering positive health outcomes in this population.

## 1. Introduction

Diabetes mellitus (DM), a complex metabolic disorder characterized by chronic hyperglycemia, is an important global health concern, ranking among the top 10 causes of death, with approximately 1.5 million annual deaths attributed to diabetes-related complications.^[1]^ The prevalence of diabetes worldwide has been on the rise, reaching an estimated 537 million adults aged 20 to 79 in 2021, and projections indicate a further increase to 643 million by 2030 and 784 million by 2045.^[1,2]^

In East African countries, including Uganda, research has consistently revealed that over 40% of diabetic patients exhibit suboptimal glycemic control, and more than 80% experience at least one complication associated with DM.^[3]^ Effective diabetes management relies significantly on blood glucose monitoring, a standard practice enabling physicians to adjust treatment strategies and minimize complications.^[4]^ Among the various clinical methods for daily diabetes management, capillary blood glucose monitoring using a glucose meter has emerged as a fundamental tool.^[5]^ The world’s first pocket-sized blood glucose meter, introduced in the late 1960s, allows for convenient and economical monitoring, with scholars noting a good correlation between values obtained from glucose meters and automatic biochemical analyzers.^[6]^

While self-monitoring of blood glucose (SMBG) has demonstrated positive outcomes in patients with type 1 DM (T1DM), its impact on patients with type 2 DM (T2DM), particularly those not using insulin, has yielded conflicting evidence.^[7]^ Studies have shown limited or no correlation between blood glucose monitoring frequency and glycemic control in this population.^[8]^ The “efficacy of SMBG in patients with newly diagnosed type 2 diabetes” trial, for instance, reported an improvement in glycated hemoglobin levels among newly diagnosed T2DM patients using SMBG, but the improvement did not reach statistical significance.^[9]^

The adoption and utilization of blood glucose meters vary across regions due to differences in economic development and the availability of DM treatment options.^[10]^ However, there is limited data on glucose self-monitoring practices among patients with T2DM on oral antihyperglycemic therapy in Uganda. Therefore, we evaluated glucometer ownership and plasma blood glucose monitoring practices among T2DM patients in a resource-limited setting in northern Uganda.

## 2. Methods

### 2.1. Study design

A facility-based, quantitative, cross-sectional study was conducted between June and August 2023, recruiting insulin-naïve T2DM patients attending routine health care at Gulu Regional Referral Hospital (GRRH), Gulu, Uganda. We followed the Strengthening the Reporting of Observational Studies in Epidemiology Guidelines.

### 2.2. Study setting

The study was conducted in GRRH, located in the northern Uganda in Gulu City. GRRH is a tertiary and a teaching Hospital serving Northern Uganda, with an estimated 2000 DM patients at the diabetic clinic. The clinic is run by a physician, medical officers, and intern doctors, with the additional workforce being derived from a pool of general and specialized nurses, lab technologists, nutritionists, and social workers. What days of the week was the clinic being run? What activities were carried out in the clinics?

### 2.3. Study population

The study participants were patients with physician-diagnosed DM2 who were 18 years or older, attending outpatient DM clinics and provided written informed consent to participate in the study. Participants who were admitted and unconscious or sedated with difficulty of communication during the study period were excluded.

### 2.4. Sample size estimation

The sample size was estimated using the Kish-Leslie formula with the following assumptions: margin of error 5%, at a 95% confidence interval (CI), the prevalence of willingness modestly estimated at 50% since there was no previous study in Uganda about willingness to start insulin therapy. Using a 10% nonresponse rate the final calculated sample size was 422. However, for this pilot study, we sought to recruit 190 study participants to inform sample size calculation for a larger cohort study.

### 2.5. Sampling procedure

We used a consecutive sampling technique to enroll eligible participants following their clinical reviews.

### 2.6. Data collection and tools

Data was collected using an interviewer-administered, structured questionnaire. It was collected by trained research assistants with regular supervision. Sociodemographic characteristics such as age, sex, religion, marital status, clinical factors, attitudes, and barriers to using insulin.

### 2.7. Operational definitions

1. Glucometer ownership was assessed using a single question: *“*Do you own a glucose monitoring device (glucometer)?” with response options “Yes” or “No” indicating the participants’ preferences.
2. Glucose monitoring was assessed through the question: “ How often do you measure your blood sugars?” with response options “At least once a week” or “Only at reviews in DM clinic” indicating the participants’ preferences.

### 2.8. Statistical analysis

Data was entered into Microsoft Excel for coding and cleaning and exported to STATA version 18 for statistical analysis. Responses with missing data were included during analysis. Descriptive statistics of variables such as age, sex, and marital status was computed in frequency, percentage, median, mean, and standard deviation and presented using tables to show the picture of the data. Bivariate logistic regression was performed to determine each of the explanatory variables with outcome variables (glucose monitoring and glucometer ownership) and variables with *P* < .2 during bivariate analysis were considered for further analysis to multivariable analysis. Poisson regression was conducted to determine the presence of a statistically significant association between explanatory variables and outcome variables. Finally, variables with *P* ≤ .05 were considered statistically significant, presented by adjusted prevalence ratio (aPR) with a corresponding 95% CI.

## 3. Results

### 3.1. Sociodemographic characteristics of patients with diabetes mellitus in Gulu, Uganda

**Figure 1.**
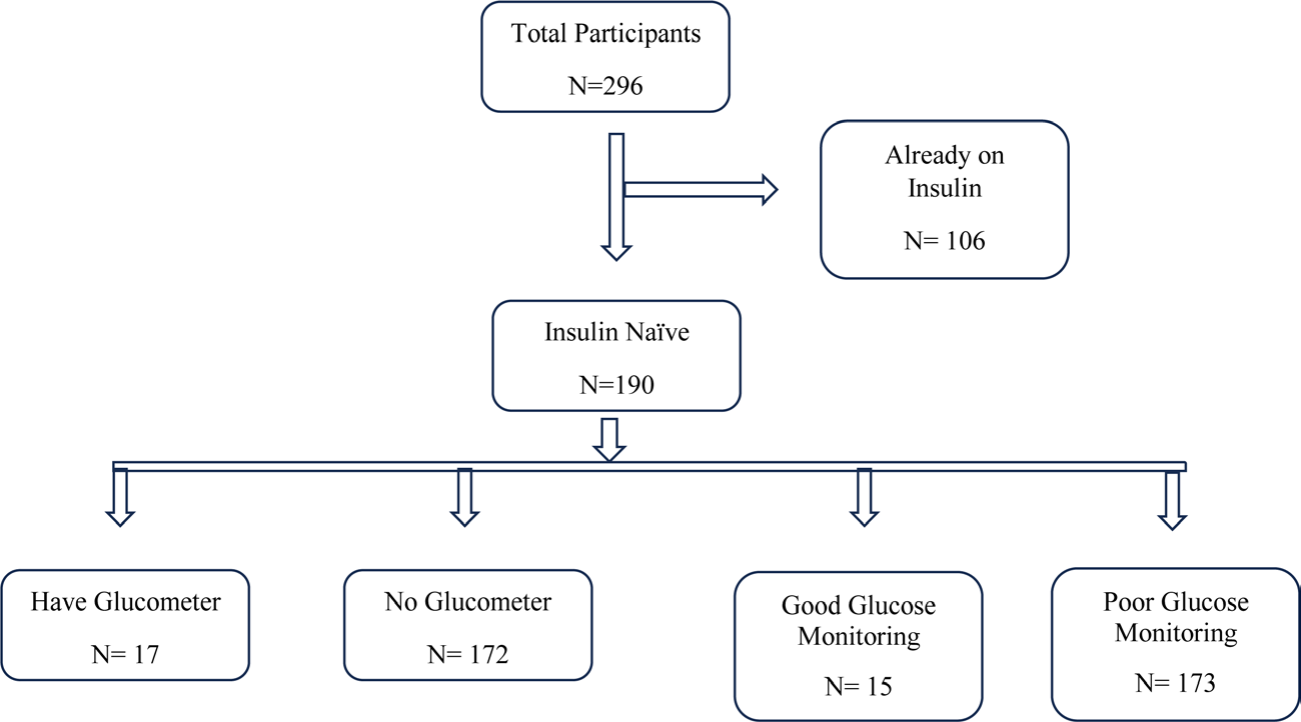
Flow diagram for the inclusion criteria for the study.

Of 296 participants screened, 106 were on insulin and were excluded and 190 were eligible (Fig. 1). The mean age of the study participants was 55 ± 12.72 years. Most participants were female (63.7%, n = 121), attained primary level of education (70.0%, n = 133), and were unemployed (84.2%, n = 160). The median household income was 50,000 Ugandan Shillings (UgX) (IQR: 50,000–150,000; 1 USD = 3750 UgX). Almost 72.5% (n = 132) of the participants earn a monthly household income between 10,000 and 100,000 UgX per month, Table 1.

**Table 1:** Sociodemographic characteristics of patients with diabetes mellitus in Gulu, Uganda.

Variable	Frequency	%
Age		
Mean (standard deviation)	55	12.72
<30	4	2.1
30–60	121	63.7
>60	65	34.2
Gender		
Male	57	30.0
Female	133	70.0
Education attainment		
Primary	84	44.2
Secondary	66	34.7
Tertiary	20	10.5
No schooling	20	10.5
Employment status		
Employed	30	15.8
Unemployed	160	84.2
Monthly household income, Ugandan shillings		
Median (interquartile range)	50,000	50,000–150,000
<10,000	4	2.2
10,000–100,000	132	72.5
>100,000	46	25.3

1 USD = 3750 Ugandan shillings.

### 3.2. Glucometer ownership and glucose monitoring practices

Most participants, 91.0% (n = 172), reported having received training on glycemic control, with only 9.0% (n = 17) reporting ownership of a glucometer, and only 8.0% (n = 15) monitored their blood sugars at least once a week (Table 2).

**Table 2:** Glucometer ownership and glucose monitoring among patients with diabetes mellitus in Gulu, Uganda.

Variable	Frequency	%
Received training on glycemic control		
Yes	172	91.0
No	17	9.0
Do you own a glucometer		
Yes	17	9.0
No	172	91.0
If yes, do know how to use is		
Yes	16	94.1
No	1	5.9
How often do you measure your blood sugars		
At least once a weak	15	8.0
Only at reviews in diabetes mellitus clinic	173	92.0

### 3.3. Diabetic characteristics of patients with diabetes mellitus in Gulu, Uganda

**Figure 2.**
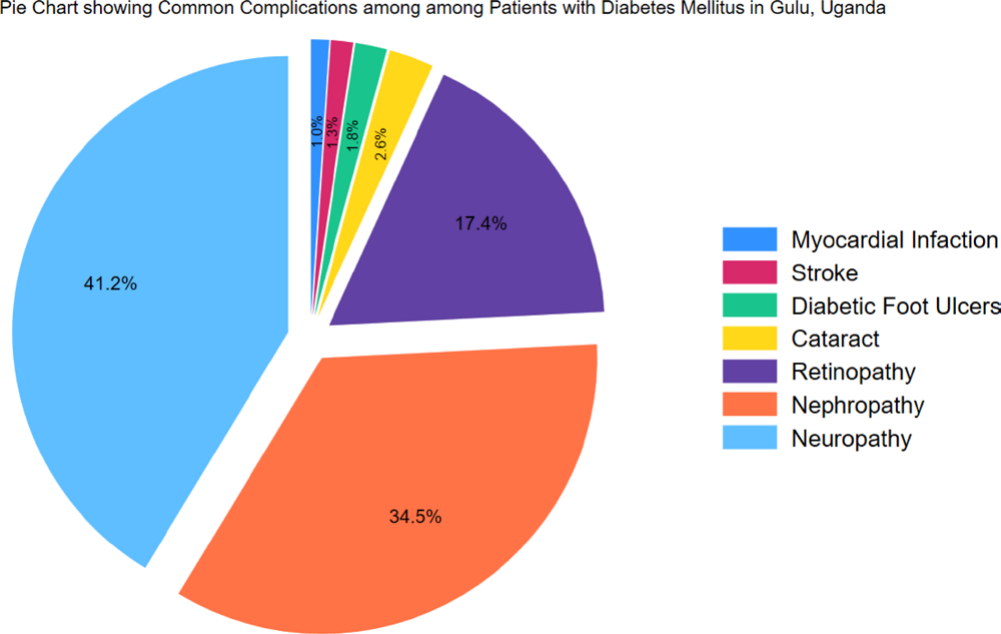
Common complications among patients with Diabetes mellitus in Gulu, Uganda.

Overall, 38.5% (n = 70) of the participants had diabetes for a period of 1 to 3 years. For treatment, 70.6% (n = 132) were on a combination of glibenclamide and metformin for DM treatment, and 91.7% (n = 121) on high blood pressure treatment. About 37.6% (n = 71) visited the clinic every month (Table 3). However, 41% of participants have neuropathy as the complication (Fig. 2).

**Table 3:** Diabetic characteristics of patients with diabetes mellitus in Gulu, Uganda.

Variable	Frequency	Percentage
Diabetes duration, years		
Median (interquartile range)	4	2–8
>1	12	6.3
1–3	70	36.8
4–6	47	24.7
>6	61	32.1
Current diabetes treatment		
Glibenclamide	10	5.4
Metformin	44	23.5
Glibenclamide and metformin	133	71.2
How often do you come for clinic visits		
Every month	71	37.6
Every 2 months	56	26.6
3 months or more	62	32.8
Do you have a comorbidity		
Yes	136	71.6
No	54	28.4
Do you have any diabetes complication		
Yes	173	91.1
No	17	8.9

### 3.4. Factors associated with glucometer ownership and glucose monitoring among patients with diabetes mellitus in Gulu, Uganda

At bivariate analysis, 9% of the participants owned a glucometer. Ownership was significantly associated with higher education levels (*P* = .007), employment status (*P* = .022), longer duration of diabetes (*P* = .031), and use of metformin as a sole treatment (*P* = .045). with 35.3% of glucometer owners had attained tertiary education compared with 8.1% of nonowners, and 35.3% were employed versus 14% among nonowners (Table 4).

**Table 4:** Bivariate analysis for factors associated with glucometer ownership among patients with diabetes mellitus in Gulu, Uganda.

Variable	Glucometer Ownership			
	All N = 189 (%)	No N = 172 (91.0)	Yes N = 17 (9.0)	*P* Value
Age				
Mean (standard deviation)	55(12.72)	55(12.01)	58(10.79)	
<30	4 (2.1%)	4 (2.3%)	0 (0.0%)	.424
30–60	121 (64.0%)	112 (65.1%)	9 (52.9%)	
>60	64 (33.9%)	56 (32.6%)	8 (47.1%)	
Gender				
Male	56 (29.6%)	51 (29.7%)	5 (29.4%)	.984
Female	133 (70.4%)	121 (70.3%)	12 (70.6%)	
Education attainment				
Primary	83 (43.9%)	78 (45.3%)	5 (29.4%)	.007
Secondary	66 (34.9%)	61 (35.5%)	5 (29.4%)	
Tertiary	20 (10.6%)	14 (8.1%)	6 (35.3%)	
No schooling	20 (10.6%)	19 (11.0%)	1 (5.9%)	
Employment status				
Employed	30 (15.9%)	24 (14.0%)	6 (35.3%)	.022
Unemployed	159 (84.1%)	148 (86.0%)	11 (64.7%)	
Monthly household income, Ugandan shillings				
Median (interquartile range)	50,000 (50,000–150,000)	50,000 (50,000–100,000)	75,000 (35,000–500,000)	
<10,000	4 (2.2%)	3 (1.8%)	1 (6.2%)	.089
10,000–100,000	131 (72.4%)	123 (74.5%)	8 (50.0%)	
>100,000	46 (25.4%)	39 (23.6%)	7 (43.8%)	
Received training on glycemic control				
Yes	171 (91.0%)	156 (91.2%)	15 (88.2%)	.682
No	17 (9.0%)	15 (8.8%)	2 (11.8%)	
Diabetes duration, years				
Median (interquartile range)	4 (2–8)	4 (2–7.5)	8(5–12)	
<1	12 (6.3%)	12 (7.0%)	0 (0.0%)	.031
1–3	70 (37.0%)	68 (39.5%)	2 (11.8%)	
4–6	47 (24.9%)	42 (24.4%)	5 (29.4%)	
>6	60 (31.7%)	50 (29.1%)	10 (58.8%)	
Current diabetes treatment				
Glibenclamide	10 (5.4%)	10 (5.9%)	0 (0.0%)	.045
Metformin	44 (23.7%)	36 (21.3%)	8 (47.1%)	
Glibenclamide and metformin	132 (71.0%)	123 (72.8%)	9 (52.9%)	
How often do you come for clinic visits				
Every month	71 (37.8%)	63 (36.8%)	8 (47.1%)	.696
Every 2 months	56 (29.8%)	52 (30.4%)	4 (23.5%)	
3 months or more	61 (32.4%)	56 (32.7%)	5 (29.4%)	

At bivariate analysis, 8% of participants reported monitoring their blood glucose at least once a week, while the vast majority (92%) did so only during clinic reviews. Frequent glucose monitoring was significantly associated with higher education (*P* = .001), employment (*P* = .008), and higher household income (*P* = .048). Specifically, 40% of weekly monitors had tertiary education compared with 8.1% of those who monitored only at clinic visits (Table 5).

**Table 5:** Bivariate analysis for factors associated with glucose monitoring among patients with diabetes mellitus in Gulu, Uganda.

Variable	Glucose Monitoring			
	Total N = 188 (%)	Only at Reviews in DM Clinic N = 173 (92.0)	At Least Once A Week N = 15 (8.0)	*P* Value
Age				
Mean (standard deviation)	55(12.72)	55(12.89)	58(11.19)	
<30	4 (2.1%)	4 (2.3%)	0 (0.0%)	.257
30–60	119 (63.3%)	112 (64.7%)	7 (46.7%)	
>60	65 (34.6%)	57 (32.9%)	8 (53.3%)	
Gender				
Male	56 (29.8%)	52 (30.1%)	4 (26.7%)	.783
Female	132 (70.2%)	121 (69.9%)	11 (73.3%)	
Education attainment				
Primary	84 (44.7%)	80 (46.2%)	4 (26.7%)	.001
Secondary	64 (34.0%)	61 (35.3%)	3 (20.0%)	
Tertiary	20 (10.6%)	14 (8.1%)	6 (40.0%)	
No schooling	20 (10.6%)	18 (10.4%)	2 (13.3%)	
Employment status				
Employed	30 (16.0%)	24 (13.9%)	6 (40.0%)	.008
Unemployed	158 (84.0%)	149 (86.1%)	9 (60.0%)	
Monthly household income, Ugandan shillings				
Median (interquartile range)	50,000 (50,000–150,000)	50,000 (50,000–100,000)	100,000 (50,000–500,000)	
<10,000	4 (2.2%)	3 (1.8%)	1 (6.7%)	.048
10,000–100,000	131 (72.8%)	124 (75.2%)	7 (46.7%)	
>100,000	45 (25.0%)	38 (23.0%)	7 (46.7%)	
Received training on glycemic control				
Yes	170 (90.9%)	156 (90.7%)	14 (93.3%)	.733
No	17 (9.1%)	16 (9.3%)	1 (6.7%)	
Diabetes duration, years				
Median (interquartile range)	4 (2–8)	4 (2–8)	7(5–8)	
>1	12 (6.4%)	12 (6.9%)	0 (0.0%)	.100
1–3	69 (36.7%)	67 (38.7%)	2 (13.3%)	
4–6	47 (25.0%)	42 (24.3%)	5 (33.3%)	
>6	60 (31.9%)	52 (30.1%)	8 (53.3%)	
Current diabetes treatment				
Glibenclamide	10 (5.4%)	10 (5.9%)	0 (0.0%)	.228
Metformin	44 (23.8%)	38 (22.4%)	6 (40.0%)	
Glibenclamide and metformin	131 (70.8%)	122 (71.8%)	9 (60.0%)	
How often do you come for clinic visits				
Every month	71 (38.0%)	63 (36.6%)	8 (53.3%)	.435
Every 2 months	54 (28.9%)	51 (29.7%)	3 (20.0%)	
3 months or more	62 (33.2%)	58 (33.7%)	4 (26.7%)	

Factors independently associated with Glucose monitoring were tertiary education (aPR: 4.48; 95% CI: 1.21–16.67; *P* = .025) (Table 6).

**Table 6:** Poisson regression analysis of factors associated with glucometer ownership and glucose monitoring among patients with diabetes mellitus in Gulu, Uganda.

Variable	Glucometer Ownership		Glucose Monitoring	
	aPR (95% CI)	*P* Value	aPR (95% CI)	*P* Value
Education attainment				
Primary	Reference	Reference	Reference	Reference
Secondary	1.32 (0.35–4.89)	.682	1.21 (0.25–5.68)	.806
Tertiary	3.83 (0.92–15.89)	.064	4.48 (1.21–16.67)	**.025**
No schooling	0.78 (0.11–5.47)	.803	1.89 (0.35–10.16)	.454
Employment status				
Employed	Reference	Reference	Reference	Reference
Unemployed	0.87 (0.19–3.91)	.855	0.75 (0.15–3.57)	.713
Monthly household income, Ugandan shillings				
<10,000	Reference	Reference	Reference	Reference
10,000–100,000	0.29 (0.03–2.73)	.285	0.27 (0.02–2.54)	.253
>100,000	0.28 (0.02–3.48)	.322	0.26 (0.02–3.10)	.282

aPR = adjusted prevalence ratio, CI = confidence interval.

## 4. Discussion

In this study, we investigated glucometer ownership and plasma glucose monitoring practices among patients with T2DM in a resource-limited setting in northern Uganda. We found low ownership of glucometers, with only 1 in every 10 person with T2DM using the device. Consequently, 9 in every 10 patients only measured their blood glucose during scheduled clinic reviews. Furthermore, over 90% of the study participants self-reported at least one complication of DM, notably hypertension in about two-thirds of the study population. Tertiary education was associated with a 4.5-fold higher likelihood of good glucose monitoring practices. These findings further confirm the high burden of complications in patients with T2DM and the need for enhanced health education on SMBG to improve glycemic control and minimize the incidence of T2DM-related complications.

The present study highlights that less than a quarter of participants (9.0%) owned a glucometer. Contrary to our findings, a study conducted in China showed a higher ownership of blood glucose meters, approximately 54.08% of participants, with statistical differences observed between those with and without glucose meters, in terms of education, occupation, chronic complications of diabetes, and knowledge of glucose control goals.^[11]^ However, in this study, there was no statistical significance in those factors. There is a need to actively increase the purchase of glucose meters among diabetic patients, by educating them about the importance of SMBG^[12]^

The findings from this study highlight a concerning lack of glucometer ownership among T2DM patients in a resource-limited setting. The majority relied solely on clinic reviews for blood sugar measurements, reflecting limited engagement in self-monitoring practices. A high percentage reported experiencing at least one diabetes-related complication, primarily neuropathy. The noteworthy association between tertiary education and improved glucose monitoring practices emphasizes the importance of educational interventions. The identified need to actively promote the purchase of glucose meters among diabetic patients through targeted education initiatives emphasizes the potential impact of comprehensive patient education on preventing complications and improving overall care for individuals with T2DM in resource-limited settings.

This study established that tertiary education was independently associated with good glucose monitoring practices. Similar findings were reported in a study in Australia where subjects with suboptimal glucose control had poor self-care practices because of their age and education level of the subjects since more than half of the participants were above 55 years and had less than 6 years of education.^[13]^ Previous studies have consistently reported a relationship between a low level of education and older subjects with poor diabetes-related knowledge on glucose control.^[4]^ Knowledgeable patients were more likely to perform self-management activities. Providing patient education about diabetes care processes should be tested as a means of increasing care to prevent diabetes complications.^[13]^

### 4.1. Study limitations

The main limitation of the study is self-report rather than direct observation of self-care practices. Other factors that limit generalizability include the use of a convenience sample and cross-sectional design. However, this study provides a baseline knowledge to inform clinical practice and education of persons T2DM on the need for home-based plasma glucose monitoring even in individuals on oral antihyperglycaemic agents.

## 5. Conclusion and recommendations

In this study, we investigated glucometer ownership and plasma glucose monitoring practices among T2DM patients in a resource-limited setting in northern Uganda. The findings from the study highlight a concerning lack of glucometer ownership and the majority relied solely on clinic reviews for blood sugar measurements. Tertiary education was independently associated with good glucose monitoring practices. The noteworthy association between tertiary education and improved glucose monitoring practices emphasizes the importance of educational interventions.

## Acknowledgments

We acknowledge all the study participants for their valuable time and support in completing the study.

## Author contributions

**Conceptualization:** Brenda Nakitto, Felix Bongomin.

**Data curation:** Brenda Nakitto, Felix Bongomin.

**Formal analysis:** Brenda Nakitto, Felix Bongomin.

**Funding acquisition:** Brenda Nakitto, Felix Bongomin, Lauryn Nsenga.

**Investigation:** Brenda Nakitto, Felix Bongomin, Ingrid Ampeire, Lauryn Nsenga.

**Methodology:** Brenda Nakitto, Felix Bongomin.

**Project administration:** Brenda Nakitto, Felix Bongomin, Ingrid Ampeire.

**Resources:** Brenda Nakitto, Felix Bongomin, Ingrid Ampeire, Lauryn Nsenga.

**Software:** Brenda Nakitto, Felix Bongomin, Winnie Kibone, Lauryn Nsenga.

**Supervision:** Brenda Nakitto, Felix Bongomin, Winnie Kibone, Ingrid Ampeire, Lauryn Nsenga.

**Validation:** Brenda Nakitto, Felix Bongomin, Winnie Kibone, Ingrid Ampeire, Lauryn Nsenga.

**Visualization:** Brenda Nakitto, Felix Bongomin, Lauryn Nsenga.

**Writing—original draft:** Brenda Nakitto, Felix Bongomin, Winnie Kibone.

**Writing—review & editing:** Brenda Nakitto, Felix Bongomin.
